# Application of Single-Nucleotide Polymorphism-Related Risk Estimates in Identification of Increased Genetic Susceptibility to Cardiovascular Diseases: A Literature Review

**DOI:** 10.3389/fpubh.2017.00358

**Published:** 2018-01-31

**Authors:** Szilvia Fiatal, Róza Ádány

**Affiliations:** ^1^Department of Preventive Medicine, Faculty of Public Health, University of Debrecen, Debrecen, Hungary; ^2^WHO Collaborating Centre on Vulnerability and Health, Department of Preventive Medicine, Faculty of Public Health, University of Debrecen, Debrecen, Hungary; ^3^MTA-DE Public Health Research Group of the Hungarian Academy of Sciences, Faculty of Public Health, University of Debrecen, Debrecen, Hungary

**Keywords:** genetic screening, genetic susceptibility, single-nucleotide polymorphism, translational research, cardiovascular diseases, literature search

## Abstract

**Background:**

Although largely preventable, cardiovascular diseases (CVDs) are the biggest cause of death worldwide. Common complex cardiovascular disorders (e.g., coronary heart disease, hypertonia, or thrombophilia) result from a combination of genetic alterations and environmental factors. Recent advances in the genomics of CVDs have fostered huge expectations about future use of susceptibility variants for prevention, diagnosis, and treatment. Our aim was to summarize the latest developments in the field from a public health perspective focusing on the applicability of data on single-nucleotide polymorphisms (SNPs), through a systematic review of studies from the last decade on genetic risk estimating for common CVDs.

**Methods:**

Several keywords were used for searching the PubMed, Embase, CINAHL, and Web of Science databases. Recent advances were summarized and structured according to the main public health domains (prevention, early detection, and treatment) using a framework suggested recently for translational research. This framework includes four recommended phases: “T1. From gene discovery to candidate health applications; T2. From health application to evidence-based practice guidelines; T3. From evidence-based practice guidelines to health practice; and T4. From practice to population health impacts.”

**Results:**

The majority of translation research belongs to the T1 phase “translation of basic genetic/genomic research into health application”; there are only a few population-based impacts estimated. The studies suggest that an SNP is a poor estimator of individual risk, whereas an individual’s genetic profile combined with non-genetic risk factors may better predict CVD risk among certain patient subgroups. Further research is needed to validate whether these genomic profiles can prospectively identify individuals at risk to develop CVDs. Several research gaps were identified: little information is available on studies suggesting “Health application to evidence-based practice guidelines”; no study is available on “Guidelines to health practice.” It was not possible to identify studies that incorporate environmental or lifestyle factors in the risk estimation.

**Conclusion:**

Currently, identifying populations having a larger risk of developing common CVDs may result in personalized prevention programs by reducing people’s risk of onset or disease progression. However, limited evidence is available on the application of genomic results in health and public health practice.

## Introduction

Despite the fact that cardiovascular diseases (CVDs) are largely preventable they are the biggest cause of death worldwide, responsible for almost one-third of all global deaths (1). In 2012, CVDs were responsible for 46% of deaths caused by non-communicable diseases. Of these deaths, an estimated 7.4 million were due to ischemic heart disease and 6.7 million were due to stroke (2). This review focuses on common complex cardiovascular disorders with high public health importance caused by a combination of several genetic and environmental factors.

The role of social determinants (e.g., aging, employment, income, and education), behavior (e.g., unhealthy diet, tobacco use, physical inactivity, and harmful use of alcohol), and metabolic (e.g., obesity, diabetes, raised blood TG, and LDL-cholesterol level, low blood HDL-C level) risk factors have largely been well known for decades due to the large-scale longitudinal studies (e.g., Framingham study and Seven Countries study) (3–8). Nevertheless, the contribution of inherited (genetic) disposition is still the focus of intense research interest (2). Our knowledge of these non-genetic risk factors has been useful in disease prevention efforts, but hopefully, we may discover more effective ways of preventing and controlling CVDs if we understand the genetics underlying these diseases.

The large majority of the common CVDs are developed as a result of harmful interaction between heritable and environmental factors. Approaches to identifying the genetic causes of polygenic common CVDs (and also other polygenic diseases) became more prominent after completion of the Human Genome Project. Several genetic loci associated with cardiovascular traits have been identified by candidate gene and genome-wide association studies testing a set of genetic variants, mainly in case–control studies in populations of different ancestry and ethnicity. Evidence for the strong contribution of genetic factors in the development of common CVDs has consistently been reported in twin and family-based linkage studies. Heritability estimates from large twin studies suggest that genetic variations may account for about 30–50% of hypertension risk and about 50–60% of coronary artery disease or myocardial infarction risk (9–12). For these complex cardiovascular disorders, the main ambition of public health initiatives is to be able to prevent or predict diseases by identification of the subject at high risk (13). In addition, several variants for monogenic subtypes of, e.g., hypertension, congenital heart disease, or familial hypercholesterinaemia have been identified (14–16). For monogenic disorders, the major public health priorities are genetic screening and its effective use in health-care practice to arrange the best treatment and provide the best care for family members at high risk (13).

Rapid advances in the genomics of CVDs have fostered huge expectations about the future use of detecting susceptibility variants in prevention, diagnosis, and treatment. Although large-scale association studies promote the estimation and categorization of the predictive values related to genetic variants, and the large number of genetic loci associated with CVDs and cardiovascular risk factors have provided insights into the biologic pathways that underlie the cases of disease, the application of such findings to cardiovascular risk prediction, prevention, and treatment still needs to be elucidated (17). So far only a small number of findings in human genome research have resulted in evidence-based applications in the field of medicine and public health.

Genetic screenings, as Becker et al. indicate, aim at populations of asymptomatic individuals, or at subpopulations in which the risk is known to be increased, or in which the specific phase of life merits screening (pregnant women, newborns) (18). Screening for common complex CVDs would give us opportunities for preventive strategies related to lifestyle, medication, or intervention (18). In the last 10 years, there has been increased enquiry into the potential clinical and public health applications of genetic screening/genetic testing of CVD risk.

In this review, we will discuss the application of single-nucleotide polymorphisms (SNPs) related to risk estimates in identification of increased genetic susceptibility to common CVDs from a public health perspective and summarize the recent advances in translational research from the past decade using the comprehensive framework suggested by Khoury and his colleagues (19). This framework comprises four phases of evidence in translation research [“T1. From gene discovery to candidate health applications; T2. From health application to evidence-based practice guidelines; T3. From evidence-based practice guidelines to health practice; and T4. From practice to population health impact” (19)] and possible future applications of genetic screening/testing of CVDs in connection with these phases can be identified.

## Methods

**Figure 1:**
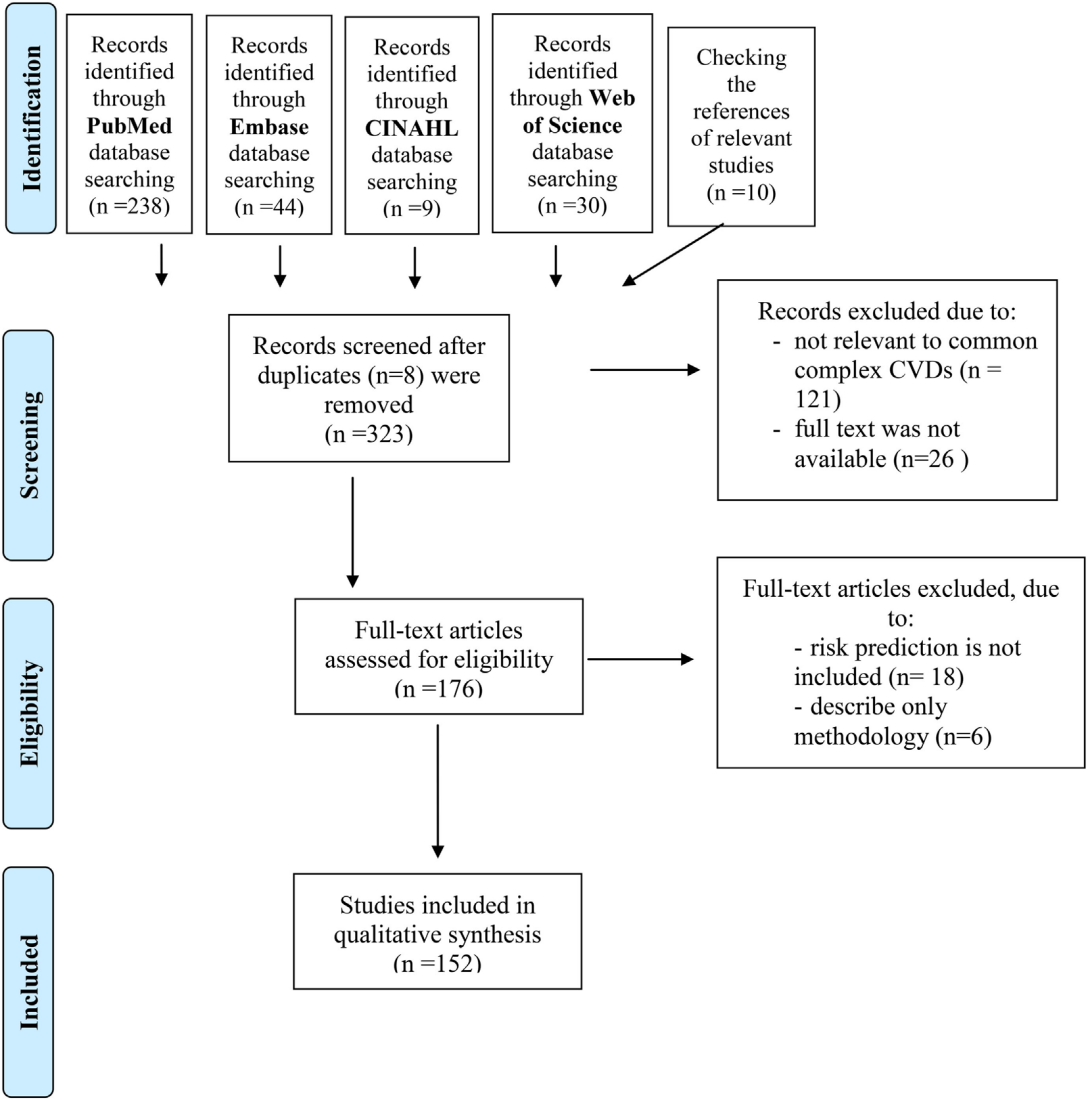
Flowchart shows study selection procedure. Adapted from Ref. (20).

To identify relevant studies in the field of CVD genetics/genomics a structured literature search using compound terms (Table 1a–d) was performed using online database services of PubMed, Embase, CINAHL, and Web of Science. The timeframe of the search related to this review was from May 5, 2007 until May 5, 2017. The systematic search and selection process were conducted as proposed in the published PRISMA guideline (20) resulting in the final list of relevant publications (see adapted flowchart, Figure 1 in Section “Results”).

**Table 1:** Details on the systematic search.

(a) Database: PubMed		
**Search number**	**MeSH Keywords used in query**	**Results**
1	((“Mass Screening”) OR “Genetic Testing”) AND “Cardiovascular Diseases”	12,735
2	(((“Mass Screening”) OR “Genetic Testing”) AND “Cardiovascular Diseases”) AND “Polymorphism, Genetic”	552
3	(((“Mass Screening”) OR “Genetic Testing”) AND “Cardiovascular Diseases”) AND “Polymorphism, Genetic”	238
	Filters activated: full text, humans, and English, published in the last 10 years	

**(b) Database: Embase**		

**Search number**	**Embase subject headings used in query**	**Results**
1	(“Mass Screening” OR “Genetic Screening”) AND “Cardiovascular Disease”	2,474
2	(“Mass Screening” OR “Genetic Screening”) AND “Cardiovascular Disease” AND “Genetic Polymorphism”	72
3	(“Mass Screening” OR “Genetic Screening”) AND “Cardiovascular Disease” AND “Genetic Polymorphism”	44
	Filters activated: full text, humans, and English, published in the last 10 years	

**(c) Database: CINAHL**		

**Search number**	**CINAHL headings used in query**	**Results**
1	(“Health Screening” OR “Genetic Testing”) AND “Cardiovascular Diseases”	69
2	(“Health Screening” OR “Genetic Testing”) AND “Cardiovascular Diseases” AND “Polymorphism, Genetic”	9
3	(“Health Screening” OR “Genetic Testing”) AND “Cardiovascular Diseases” AND “Polymorphism, Genetic”	1^a^
	Filters activated: full text, humans, and English, published in the last 10 years	

**(d) Database: Web of Science**		

**Search number**	**Keyword used in query**	**Results**
1	(“Mass Screening” OR “Genetic Testing”) AND “Cardiovascular Diseases”	217
2	(“Mass Screening” OR “Genetic Testing”) AND “Cardiovascular Diseases” AND “Polymorphism, Genetic”	38
3	(“Mass Screening” OR “Genetic Testing”) AND “Cardiovascular Diseases” AND “Polymorphism, Genetic”	30
	Filters activated: full text, humans, and English, published in the last 10 years	

*Strategy of search: date of query: May 5, 2017*.

*Medical Subject Headings (MeSH) is the United States National Library of Medicine’s controlled vocabulary thesaurus used for indexing articles for PubMed*.

*^a^Full text was available in case of 1 article*.

## Results

### Studies Included in the Review by Implementing PRISMA Statement

The first step was *the identification of the records*. A primary search using PubMed, Embase, CINAHL, and Web of Science was performed to identify related publications using compound terms (Table 1a–d). Research communications published earlier than 2007 and not published in English were excluded. Only those studies that were conducted on human subjects and were available in full-text format were considered. This identification step resulted in 323 records. Moreover, by checking the references of the relevant studies an additional 10 studies were identified and included. The details of the search and the number of records identified in queries can be seen in Figure 1.

Next, the abstracts of the records that fit the abovementioned criteria were tested (*screening of the records*) for relevance to the topic, i.e., only those records were included that focused on common CVDs. Twenty-six studies were discarded because the full text of the research was not available, and 121 studies were excluded after reviewing the abstracts because it appeared that these papers clearly were not relevant to common complex CVDs.

Finally, *eligibility and inclusion* of the records were investigated: the full text of the remaining 176 publications was examined in more detail. A total of 152 studies were identified for inclusion in this review, after 24 studies were excluded because risk prediction was not included in those articles or only the methodology was described.

### Translational Genomic Research in CVDs

During the last decade, voluminous research aimed at incorporating cardiovascular genetic/genomic discoveries into practice has been undertaken. Altogether, 152 studies were found to be relevant to this field. Khoury et al. suggested a framework for translational research that is required before applying genomic findings in clinical or public health practice. This framework includes the following four phases:

T1. From discovery to candidate health applications,
T2. From health application to evidence-based practice guidelines,
T3. From practice guidelines to health practice, and
T4. From practice to population health impact (19).

The major developments on CVDs’ genomics for each phase of the framework are summarized in Table 2. Consequently, this table also displays how close/far cardiovascular genetic applications are to/from clinical or public health practice.

**Table 2:** Advances in genomic research on common cardiovascular diseases (CVDs) according to the translation research framework.

	T1 phase: discovery to candidate health application	T2 phase: health application to evidence-based practice guidelines	T3 phase: guidelines to health practice	T4 phase: practice to population health impact
Genome-based prediction of common CVDs	– Single-gene associations – Genome-wide associations – Prediction models using genetic and non-genetic factors	– Clinical validity, utility investigation to assess risk in the general population	None	None

Genetic testing to improve diagnostic accuracy	– Diagnostic models using genetic and non-genetic factors	None	None	None

Genetic testing to improve prognostic accuracy	None	None	None	None

Genome-based prediction of treatment response	– Genetic profiles – Interaction between genetic factors and response to treatment	– Clinical validity, utility investigation to predict response in high-risk group	None	– Decision-analytic model estimating cost-effectiveness (only simulation study)

#### From Gene Discovery to Candidate Health Applications (T1)

Khoury et al. describe T1 research as follows: it begins after gene discovery, and its goal is the development of candidate health applications to be used in clinical and public health practice. Ideally, the outcome of this phase is the development of a single-gene test or construction of a genome profile that has high sensitivity, specificity, and predictive value. These applications can be used to foster clinical evaluation (predictive testing, screening, diagnostic, and prognostic testing) or selection of the most effective therapeutic option, usually by observational studies and clinical trials (phases I and II) (19).

##### Genome-Based Prediction of CVDs

###### Genetic Association Studies on SNPs

During the last 10 years, numerous association studies were performed in the field of cardiovascular genetics with a high representation of case–control design among them. These studies utilized a single candidate-gene approach almost without exception to estimate the potential cardiovascular risk. Few of them were genome-wide association studies examining SNPs across the genome, and only one linkage study and one segregation study were found (21–23).

Genetic alterations were associated with ischemic stroke (24–41), arrhythmias (42–51), coronary heart/artery disease (52–62), myocardial infarction (63–66), and carotid sclerosis (67–70) were intensely investigated, but less attention was paid to the following traits/phenotypes as an outcome: atrial fibrillation (71), abdominal aorta aneurysm (72), carotid intima-media thickness (73, 74), carotid plaque thickness (75), cardiovascular mortality/diseases in general (76–80), dilated cardiomyopathy (81, 82), venous thrombosis (83–85), familial hypercholesterinaemia (86), hypertrophic cardiomyopathy (87–89), plasma lipoproteins (90), hypertension (91–93), intracranial aneurysm (94, 95), intracerebral hemorrhage (96–98), ischemic heart disease (99), recanalization after ischemic stroke (100), subarachnoid hemorrhage (101–103), vasodilator reactivity (104), and lower extremity artery disease (105). During recent years, a number of review articles have appeared dealing with recent advances in genetics research (mostly reviews of association studies) of arrhythmia (106–108), coronary artery/heart disease (78, 109–114), sudden cardiac death (115, 116), sporadic heart failure (117, 118), cardiomyopathy (119), and thrombophilia (120, 121), that also discuss the recent and potential developments in the field (122–127).

Genetic association studies investigate a correlation between disease status and a genetic alteration(s) (e.g., SNPs, VNTRs, and CNVs) to identify risk or protective alleles that play a part in the development of a specific disease. An increased frequency of a risk allele or genotype in the individuals affected with a disease can result in the conclusion that the variant of interest increases the risk of a specific disease (128). According to our results, association studies still represent an important tool in identifying genes contributing susceptibility to several complex CVDs. Association studies (and meta-analyses) have reconfirmed that many different genetic variants affect disease risk, but each variant has only a relatively small effect. Single markers identified are unlikely to be considered for clinical use unless they yield a high effect size (characterized by odds ratio/beta coefficient).

Meta-analyses that combine the results of single-gene association studies provide an opportunity to obtain more robust effect sizes. In the last decade, meta-analyses were related to atrial fibrillation, MRI-defined brain infarct, ischemic stroke, and susceptibility to any type of atherosclerotic CVD, such as coronary artery disease, acute coronary syndrome, or ischemic heart diseases (129–135). It is worth mentioning that meta-analyses may be biased: publication bias, population stratification, control selection bias, and lack of genotype blinding exist, thus results should be interpreted with caution.

###### Genome-Wide Association Studies

Genome-wide association studies use high-throughput genotyping technologies to assay thousands of SNPs and correlate them to clinical conditions or measurable traits. GWA studies are very useful in discovering genetic variants related to different diseases but also have important limitations (summarized by Pearson and Manolio), “including false-positive and false-negative results and biases related to selection of study participants, and genotyping errors.” But most of the variants identified by GWA studies still have very modest effects on disease risk and explain only a small fraction of population risk or total estimated heritability (136).

It is important to point out that a variant with even small odds ratios can improve the indicative power of the predictive models, such as the 9p21 locus (114, 137–141). Despite several studies that show consistent associations of 9p21 locus with CVD traits, the biological role of the locus is still not well understood. In a study by Visel et al. (142), the results provide direct evidence that the coronary artery disease risk interval has a crucial role in regulation of cardiac Cdkn2a/b expression (a mouse ortholog of the 9p21 locus) and suggest that this region has an effect on the progression of coronary artery disease by modifying the dynamics of vascular cell proliferation. If it is confirmed this would represent a new mechanism for myocardial infarction that is unrelated to traditional risk factors (123).

###### Combining Candidate-Gene SNPs with or without Traditional Clinical Risk Factors—The Genetic Risk Score (GRS) Approach

Multiple markers with small effect sizes may be used in combination to generate high effect size. The simultaneous use of the most common and strongest risk markers (with or without other non-genetic traditional risk factors) may have the desired discriminatory accuracy (quantified by the *C*-statistics) to distinguish between diseased and healthy subjects. Information obtained from SNPs can be combined by first assigning a risk value of, e.g., 0 for a subject that is a non-carrier of risk allele, 1 if a “carrier,” or 2 if homozygous for that allele, and then calculating the overall score (GRS) for each individual in the study population (143).

Using this gene scoring approach, Aulchenko et al. (144) included a total of 17,797–22,562 persons, aged 18–104 years from the Nordic countries to Southern Europe. They investigated 22 loci known to be associated with serum lipid levels (total cholesterol, low-density lipoprotein, cholesterol, high-density lipoprotein cholesterol, and triglycerides). GRSs based on lipid loci explained 4.8, 3.4, and 3.0% of age-adjusted variances in HDL-C, LDL-C, and TG, respectively and were also associated with increased intima-media thickness (*p* = 0.001) and coronary heart disease incidence (*p* = 0.04). They tested for the association between the genetic risk profiles and intima-media thickness, and incident coronary heart disease. From the risk profiles, the total-cholesterol profile and the combined profile—including all associated SNPs of the four traits—were most strongly associated with the clinical outcomes. They concluded that the genetic profiles developed improve the identification of subjects at high risk of dyslipidemia but do not improve the prediction of atherosclerosis and CHD compared to classical risk factors.

In the study of Krarup et al. (145), the GRS of 45 risk variants was involved to estimate the effect on incidence and clinical predictability of myocardial infarction and coronary artery disease in 6,041 Danish individuals. Analyses using two different models (model 1: adjusted for age and sex; model 2: adjusted for age, sex, BMI, smoking status, and type 2 diabetes mellitus) detected allele-dependent association of GRS with myocardial infarction [hazard ratio (HR) (95% CI): model 1: 1.05 (1.01–1.10), *p* = 0.02 and model 2: 1.06 (1.02–1.11), *p* = 0.01]. No association with coronary artery disease was shown for either GRS model. They aimed to estimate the predictive capacity of GRS for only myocardial infarction, but no significant effect was identified on discriminative or reclassification ability by adding GRS to the European SCORE algorithm (age, sex, smoking status, systolic blood pressure, and total cholesterol).

In the recent study of Isaacs et al. (146), the cumulative effects of common genetic variants related to TC, LDL-C, HDL-C, and TG were associated with carotid plaque formation. As Isaacs et al. concluded, the relationship was the strongest for the LDL-C score, which increased plaque score by 0.102 per SD increase in GRS (*p* = 3.2 × 10^−8^). TC and LDL-C scores were significantly associated with incident myocardial infarction and coronary heart disease with HRs between 1.10 and 1.13 per SD increase in score. The Framingham risk score (FRS) discriminated myocardial infarction better than the GRSs (area under receiver-operating characteristic curve—AUC 0.65 vs. 0.62); after combining FRS and GRSs, the results improved slightly compared with the FRS AUC alone (AUC 0.66; *p* = 0.069). In cases of coronary heart disease, the results were similar. In conclusion, GRSs did not improve clinical AUCs.

Tikkanen et al. (147) genotyped 28 genetic variants in a Finnish cohort of 24,124 participants. A multilocus GRS was constructed, and its association with incident CVD events was evaluated. They reported that by adding genetic information to conventional risk factors the risk discrimination of coronary heart disease (*C*-index 0.856 vs. 0.851, *p* = 0.0002) and other end points (CVD: *C*-index 0.840 vs. 0.837, *p* = 0.0004; acute coronary sclerosis: *C*-index 0.859 vs. 0.855, *p* = 0.001) were improved. According to their model in a population of 100,000 individuals, additional genetic screening of subjects at intermediate risk for coronary heart disease would reclassify additional 2,144 subjects (12%) into the high-risk category.

According to the study by Weijmans et al. (148) in a group of patients (5,742 individuals) with symptomatic vascular disease, the GRS did not improve prediction of 10-year risk of cardiovascular events beyond clinical characteristics. The net reclassification index improved only in case of patients suffering from stable atherosclerosis (0.14, 95% CI: 0.03–0.25).

Ganna et al. (149) used data from 6 Swedish prospective cohort studies with 10,612 healthy participants. They investigated the clinical utility of GRS in primary prevention of CVDs. Several risk scores were developed: the overall GRS based on 395 SNPs was reported as being associated with cardiovascular traits: one coronary heart disease-specific GRS, including 46 SNPs, and 6 trait-specific GRS for each established CHD risk factor (body mass index, HDL-C, systolic blood pressure, TC, and smoking, type 2 diabetes mellitus). The overall and the coronary heart disease-specific GRS were significantly associated with CHD risk (HRs for fourth vs. first quartile, 1.54 and 1.52; *p* < 0.001) and improved risk classification beyond established risk factors (net reclassification improvement, 4.2 and 4.9%; *p* = 0.006 and 0.017). Discrimination improvement was modest (*C*-index improvement, 0.004).

##### Genetic Testing to Improve Diagnostic Accuracy

Besides the several studies mentioned earlier on coronary heart disease, a study on risk models that predict a person’s risk for developing venous thrombosis was published by de Haan et al. (150). GRS based on 31 venous thrombosis-associated SNPs was developed for subjects of a large case–control study (2,712 patients and 4,634 controls). GRS computed from all the 31 SNPs or from the 5 most strongly associated SNPs performed very similarly (AUCs of 0.70 and 0.69, respectively). The AUC of a risk model based on known non-genetic risk factors was 0.77 (95% CI: 0.76–0.78). After combining the non-genetic and genetic risk models, the AUC improved to 0.82 (95% CI: 0.81–0.83), which indicates better diagnostic accuracy.

##### Genome-Based Prediction of Treatment Response

In addition to genetic testing that can improve the treatment by increasing drug efficacy and safety, a genetic test can be used to select patients for therapies that target-specific genes/gene products (151). An area where genome-based prediction of treatment response is important is the use of genetic testing for evaluating the antiplatelet effects of the antiplatelet drug clopidogrel. Several recent studies suggest that therapeutic responses to clopidogrel might depend on the genotype at the *CYP2C19* gene; however, some findings are contradictory (152–156). It was shown that clopidogrel-treated patients who had an allele of *CYP2C19* with reduced function [most commonly the CYP2C19*2 or CYP2C19*3 allele, roughly 30% of patients have loss-of-function (LOF) alleles] had less platelet inhibition, and consequently, a significantly higher risk of cardiovascular events than those who had a normally functioning CYP2C19 enzyme (157). Furthermore, it was shown that the CYP2C19*17 variant is the gain-of-function (GOF) allele (prevalence is between 3 and 21%) and is an independent factor in increased bleeding risk (157). Recently, Shen et al. (158) demonstrated the explicit clinical benefit of CYP2C19 genetic testing for guiding the antiplatelet therapy on a sample of 628 patients; clinical outcomes were analyzed at 1, 6, and 12 months after discharge. Individual antiplatelet therapy guided by CYP2C19 genetic testing significantly improved the prognosis of patients after percutaneous coronary intervention. The morbidity rates of “major adverse cardiovascular events” in the intervention group were decreased by 4.3, 4.6, and 5.2% compared with the routine group (conventionally treated with 75 mg daily of clopidogrel without CYP2C19 genetic testing) at 1, 6, and 12 months, respectively.

According to a review by Chan et al. (159), there is a good evidence of analytical validity for testing LOF polymorphisms in managing clopidogrel therapy. They highlighted that LOF polymorphisms are associated with reduced levels of the active clopidogrel metabolite and with reduced on-treatment inhibition of ADP-induced platelet activation. In percutaneous coronary intervention populations, there is consistent evidence for an association between LOF polymorphisms and adverse clinical outcomes (stent thrombosis and major adverse cardiovascular events). Evidence for clinical utility of CYP2C19 genotyping as a predictive biomarker is limited to subgroups with indecisive findings.

In a single-center study of 535 ischemic stroke patients who received clopidogrel, Yi et al. (160) found that for patients carrying the reduced function *LOF* polymorphisms the inhibition of platelet aggregation was significantly lower in patients treated with proton-pump inhibitors.

#### From Health Application to Evidence-Based Practice Guidelines (T2)

The second phase (T2) begins if there is convincing evidence on genetic test performance. In this phase, the so-called ACCE components (*a*nalytic and *c*linical validity, *c*linical utility and *e*thical, legal, and social issues) are investigated in the population settings for which the tests are intended. These evaluations depend on multidisciplinary research in the field of clinical medicine, laboratory sciences, economics, public health, ethics, behavioral, and social sciences. Results from this phase should result in evidence-based guidelines for both clinical and public health practice (19).

According to the Evaluation of Genomic Application in Practice and Prevention Working Group (EWG), testing for the 9p21 genetic variant or 57 other variants in 28 genes is not recommended to assess risk for CVD in the general population, specifically heart disease and stroke (161). The EWG highlighted that even if the 9p21 variants with heart disease had convincing evidence of per allele–odds ratio of between 1.2 and 1.3 (the highest among all variants they investigated) the magnitude of net health gain from use of any of these test (alone or in combination) is irrelevant. According to the guideline of the U.S. Preventive Services Task Force, genetic/genomic markers are not included among those non-traditional risk factors suggested in coronary heart disease risk assessment (carotid intima-media thickness and high sensitivity C-reactive protein) (162).

To date, several research studies have focused on the *CYP2C19* gene (see relevant studies above) because its variants can reduce the formation of the active metabolite of clopidogrel and influence clopidogrel’s antiplatelet effects. While many studies showed that clopidogrel’s efficacy depends on CYP2C19 genetic polymorphisms, others did not find any association (163, 164). In fact, CYP2C19 LOF alleles account for only 12% of the variability in response to clopidogrel. This implies that most of the variabilities are caused by other factors not yet developed. To date, guidelines form the American College of Cardiology Foundation/American Heart Association recommended against routine genetic testing in patients with acute coronary syndrome (165). In addition, a IIb recommendation (evidence C) has been given to CYP2C19 genotyping by stating that: efficacy is less well established; only diverging expert opinion and case studies are available.

The MTHFR enzyme catalyzes the transition of 5,10-methylenetetrahydrofolate to 5-methyltetrahydrofolate, the primary circulatory form of folate, and a cosubstrate for homocysteine remethylation to methionine. MTHFR polymorphism testing (for variant of c.665C → T and c.1286A → C) is frequently suggested by general practitioners as part of the clinical investigation for thrombophilia. The potential associations between *MTHFR* genotype status and several complications have been evaluated by case–control, cohort, Mendelian randomization, and meta-analysis because formerly it was suggested that reduced enzyme activity of MTHFR led to hyperhomocysteinemia, which amount to an increased risk for venous thromboembolism, coronary heart disease, and recurrent pregnancy loss (166). But a later meta-analysis has found that the association was not as strong as previously believed. Long and Goldblatt highlighted that “homozygosity for the 677C>T polymorphism is linked to a small increase in homocysteine levels; the increased risk of ischemic heart disease and stroke is more closely related to the serum levels of homocysteine rather than the presence of the MTHFR polymorphisms. Furthermore, there seems to be no increased risk of mortality from CVD to MTHFR 677C>T homozygotes (167).” Considering the fact that MTHFR polymorphism is only one out of many other factors contributing to the clinical picture, the utility of this testing is presently doubtful. There is growing evidence that MTHFR polymorphism testing has minimal clinical utility and therefore should not be prescribed as part of a routine evaluation for thrombophilia according to the American Congress of Obstetricians and Gynecologists, the American College of Medical Genetics and Genomics, and the British Society for Haematology (166–169).

Although the ethical, legal, and social issues related to genetic screening/testing of CVDs are rather important components of T2 research, they were not the focus of our interest.

#### From Evidence-Based Guidelines to Health Practice (T3)

The third phase addresses the spread and integration of knowledge yielded through the T2 phase research. The translation and dissemination of evidence-based guidelines into practice is challenging (19), and only one genomic application is ready for implementation in routine daily practice. The U.S. Preventive Services Task Force suggest BRCA mutation testing for predicting breast and ovarian cancers for women who have blood relatives with breast, ovarian, tubal, or peritoneal cancer (170), but no recommendations (published or in progress) are available regarding genetic testing of CVDs.

#### From Practice to Population Health Impact (T4)

This phase assesses how the adopted recommendations and guidelines make an impact. It focuses on clinical and public health outcomes of the guidelines obtained and includes measuring the incidence of the disease, quality of life indicators, clinical decision modeling, and cost-effectiveness analysis (19).

So far, only one study by Jiang and You (171) examined the clinical and economic outcomes of CYP2C19 LOF- and GOF-guided antiplatelet therapy in subjects with acute coronary syndrome undergoing percutaneous coronary intervention. They designed a lifelong decision-analytic model in a hypothetical cohort of 60-year-old patients to simulate outcomes of three strategies: clopidogrel, alternative P2Y12 inhibitors (prasugrel/ticagrelor), and LOF/GOF-guided therapy (LOF/GOF allele carriers received an alternative P2Y12 inhibitor and wild-type patients received clopidogrel). Direct costs, clinical event rates (including major cardiovascular events, stent thrombosis, and major bleeding), and quality-adjusted life-years gained were the model’s outcomes. They found that non-fatal myocardial infarction (5.62%) and stent thrombosis (1.2%) had the lowest rate in the alternative P2Y12 inhibitor arm, whereas non-fatal stroke (0.72%), cardiovascular death (2.42%), and major bleeding (2.73%) were the lowest in the LOF/GOF-guided group. The LOF/GOF-guided arm had the highest QALYs (7.5301 QALYs) at the lowest lifelong cost (USD 76,450). These finding suggest that personalized antiplatelet therapy driven by *CYP2C19 LOF* and *GOF* alleles appears to be the preferred antiplatelet strategy when compared to clopidogrel and alternative P2Y12 inhibitor therapy.

### Quality Assessment of Studies Included

The full text of the articles (genome-wide association studies, genetic risk prediction studies, and meta-analyses) was screened, and data on recently published key reporting components (according to STREGA, GRIPS, and PRISMA) and methodological components (according to AMSTAR) of the studies were extracted (20, 172–174). The STREGA, GRIPS, PRISMA, and AMSTAR checklists have 22, 25, 27, and 11 items, respectively, that should be reported in the research articles. If each item is achieved, the maximum scores are identical to the item numbers. The mean STREGA score of all collected GWAs was 20.8 ± 1.64 (on average, 94.54% of the items properly reported). The per item STREGA analysis revealed that one item (item 19: discussing limitations of the study) was the least adhered to (40%) of the five GWAs included. The mean GRIPS score of all genetic risk prediction studies included was 21.43 ± 2.51 (85.72% of items were reported satisfactorily). The per item GRIPS analyses showed that one item had less than 57% adherence (item 14: report the number of individuals at each stage of the study) out of the seven risk prediction studies involved. Two items (item 10: specify the procedure and data used for validation of the risk model, and item 19: report any validation of the risk model) had 28.6% adherence. The overall mean PRISMA score of meta-analysis was 22 ± 4 (82.96% of the items adequately reported). The per item PRISMA analysis showed that one item (item 5: indicate if a review protocol exists and where it can be accessed) had 0% adherence out of the five meta-analyses included. Item 25 (discuss limitations at study and outcome level) has only 40% adherence. The mean AMSTAR score of meta-analyses was 6.2 ± 1.48 (56.36% of the items adequately reported). The per item AMSTAR analysis showed that three items (item 1: Was an *a priori* design provided? Item 4: Was the status of the publication—i.e., gray literature—used as an inclusion criterion? Item 5: Was the list of studies, included and excluded, provided?) had 0% adherence out of the five meta-analyses included. Based on our analyses the reporting qualities of recently published studies were good in general, although according to the per item analyses there is a need for improvement in the case of some items. Two meta-analyses (129, 133) were excluded from PRISMA and AMSTAR analyses owing to the fact that both were meta-analyses of genome-wide association studies developed by consortia and data were not obtained *via* systematic literature search.

## Discussion

We have collected and reviewed the published literature according to an overarching framework for translational research recommended by Khoury et al. (19). The studies reviewed in this work represent the majority of current data available on genetic testing/screening for CVD risk. The relevant research papers were retrieved using a PubMed search and included original scientific papers, reviews, meta-analyses, and editorials. We have summarized the major findings of the research identified in each translation phase.

The overview of most significant outcomes in genetic research in common CVDs according to the framework for translational research is summarized in Table 3. The vast majority of the studies published relate to T1 research (24–127, 137–160), many fewer focus on T2 research (161–169); however, T3 research is missing, and only one simulation study (171) was identified as a part of the T4 research (Tables 2 and 3). These finding suggest that during the last 10 years very few cardiovascular genetic discoveries have led to evidence-based applications for medical or public health practice. Genetic prediction of the complex CVDs consists of multiple genes added to traditional risk factors (145, 146, 149). Several recent studies suggest that polymorphisms, mainly in candidate genes, may help to distinguish among several clusters/subgroups of patients. Several studies have identified certain risk profiles based on clusters of genes related to coronary heart disease or deep vein thrombosis but with low predictive values (147, 150). Further research is still needed to validate whether these genomic profiles can prospectively identify individuals at risk to develop CVDs.

**Table 3:** Overview of most significant outcomes in genetic/genomic research in common cardiovascular diseases (CVDs) according to the framework for translational research.

	T1 phase: discovery to candidate health application	T2 phase: health application to evidence-based practice guideline	T3 phase: guidelines to health practice	T4 phase: practice to population health impact
Genome-based prediction of common CVDs	– Numerous genetic alterations were associated with numerous phenotypes in single-gene studies (24–127) – 9p21 locus shows powerful association with coronary heart disease, myocardial infarction in several genome-wild associations (114, 137–141) – Total-cholesterol risk profile (based on 11 SNPs) improves identification of subjects at high risk of dyslipidemia (144) – Combining Framingham risk score and genetic risk score (GRS) (based on 336 SNPs related to TC, LDL-C, HDL-C, and TG) slightly improves clinical accuracy (146) – GRS (based on 28 variants) improves the risk discrimination of coronary heart disease over and above traditional risk factors (147) – Overall GRS (computed from 395 variants) increases risk classification of coronary heart disease beyond established risk factors (149)	– Evaluation of *Genomic Applications in Practice and Prevention Working Group* does not recommend testing for 9p21 genetic variant or other 57 SNPs in 28 genes to assess the risk of heart disease and stroke (161)	None	None

Genetic testing to improve diagnostic accuracy	– Combining GRS (computed from 31 SNPs) and non-genetic risk factors increases the diagnostic accuracy of venous thrombosis (150)	None	None	None

Genetic testing to improve prognostic accuracy	None	None	None	None

Genome-based prediction of treatment response	– Antiplatelet therapy guided by CYP2C19 gene testing for loss-of-function/gain-of-function (GOF) alleles improves cardiovascular prognosis (157, 158)	– MTHFR genetic testing for *677C*>*T* homozygosity has minimal clinical utility, not recommended as a part of routine evaluation for thrombophilia (161, 166–169)	None	– Loss-of function/GOF-guided personalized antiplatelet therapy has the highest quality-adjusted life-years at lowest lifelong cost (simulation study) (171)

The most important limitation of current cardiovascular T1 research is that only single-gene variants or several SNPs contributing to a small proportion of the genomic variation are investigated, but there are already prediction models available involving more complex system biology in large-scale and well-designed studies (144–150). We have also identified various research gaps including the following: little information is available on studies suggesting “Health application to evidence-based practice guidelines”; no study is available on “Guidelines to health practice.” Furthermore, it was not possible to identify studies that incorporated environmental or lifestyle factors into the risk estimation.

Single-gene association reports from the last decade clearly point out that the SNPs associated with increased or decreased cardiovascular risk have little impact in risk estimation. In a study reviewed, the results of more than 600 studies and 3,000 SNPs related to CVDs, the largest OR for the association between an SNP and cardiovascular trait was 1.6, and almost all OR were between 0.8 and 1.2 (175). These so-called “common variants with small effects” can explain only modest amount of heritability even if hundreds of genetic factors are used for risk prediction (176, 177). Beside these variants numerous “rare alleles” (population frequency is less than 0.5%) expected in the human genome. Rare variants have remarkable effect size and consequently might underlie the missing heritability of complex CVDs (178). To capture rare variants the GWAs approach or linkage study design are not powerful (178, 179); instead of genotyping a list of variants it is inevitable to sequence entire genome. The previous sequencing technology (known as “automated Sanger sequencing”) was expensive and time-consuming, but over the past decade, new high-throughput technologies, referred to as next-generation sequencing (NGS), were evolved. NGS technologies are cost-effective, able to explore the human genome in reasonable time and are suitable to discover full spectrum of sequence variations (180). Several recent studies suggest that the application of NGS technology in defining and characterizing inheritable components of CVDs is getting important (181–183). This strategy was successfully applied to find new genetic variants for Mendelian CVDs such as hypertension (*KCNJ5, KLHL3* gene), dilated cardiomyopathy (*BAG* gene), or familial combined hypolipidemia (*ANGPTL3* gene) (184–187). However, utilizing NGS technologies to discover novel variants contributing common CVDs is very challenging mainly because of stringent statistical requirements. Currently, this approach represents a field has not been widely explored (188, 189). A viewpoint paper from the European Society of Cardiology working group on myocardial and pericardial diseases and the members of the European Society of Human Genetics summarize their current opinion on the next-generation DNA sequencing. In routine care of patients whole exome/genome sequencing can be used as a diagnostic tool but only in case of recognized Mendelian disease genes (e.g., inherited cardiomyopathies, channelopathies, and familial dyslipidemias) (190–192). The collaboration between cardiologist, geneticist, molecular biologist, and bioinformatician are necessary in interpretation of sequencing results (190). All the challenges, advantages, and disadvantages of the NGS approach are beyond the scope of our wok.

Common CVDs are complex disorders in which gene–gene and gene–environment interactions play an important role. To date, the diagnosis of CVDs is mainly based on clinical signs and symptoms; however, the expectations surrounding genomic discoveries that improve presymptomatic testing, diagnosis, and treatment are huge. Genetic screening, a possible tool for disease prevention, is defined by the use of a set of diagnostic tests on a population to identify people who are carriers of specific genetic disorders, and who are consequently at higher risk of developing a certain disease. In the case of some common cardiovascular disorders, alteration in a single gene strongly affects the risk of development (these are referred to a “monogenic diseases,” e.g., familial hypercholesterinaemia, Mendelian forms of low and high blood pressure); however, in the case of the majority of common disorders, several genes, environmental factors, and also interactions between genes and between genes and environmental factors (considered “complex diseases,” e.g., coronary heart disease and venous thromboembolism) are required (188).

Smoking, harmful alcohol consumption, physical inactivity, and unhealthy eating habits are the most significant preventable behavioral risk factors of CVDs. As illustrated by several studies, gene–gene and gene–environment interactions contribute to the initiation and maintenance of these risk behaviors, which in turn increases risk for CVDs (193–196). Investigations of genetic and environmental attributes associated with ethnicity are an essential component of multidisciplinary research into the prevention of diseases, including those that differ in prevalence among ethnic groups such as CVDs (197, 198). The Roma population, which constitutes the largest ethnic minority in Europe, is the main subject of ethnicity-based studies because available data strongly suggest that Roma populations suffer from poorer health and lower life expectancy (199). Recently, our research group investigated whether genetic susceptibility contributes to the higher prevalence of smoking, harmful alcohol drinking habits and reduced HDL-C level in the Roma population compared to the general Hungarian population (200–202). Estimating the extent of genetic susceptibility might be important for designing and implementing targeted public health intervention programs among Roma. According to our results, harmful health behaviors (smoking and alcohol consumption) among Romani people have environmental/cultural underpinnings rather than inheritable attributes thus interventions aimed at smoking and alcohol consumption should preferentially target the cultural/environmental factors. However, in the case of reduced HDL-C levels, the contribution of genetic susceptibility was confirmed hence interventions aimed at this risk factor need to consider the increased genetic susceptibility of Roma.

Genetic testing offered for single-gene disorders known to be associated with CVDs (e.g., familial hypercholesterinaemia or Tangier disease) (18, 192). Presently, genetic screening is recommended for high-risk groups only in special cases (for example, cascade testing from known cases of Lynch syndrome and familial hypercholesterinaemia or testing for women at high risk of breast cancer because of their family history). In contrast with single-gene disorders, screening is limited for estimating susceptibility to multifactorial CVDs (203).

Caution is required before spreading out the use of such genetic screening tests to population level, because the positive predictive value of any variants found is low, and it would be hard to interpret the findings (13). Furthermore, several fundamental questions raised by Thanassoulis and Vasan concerning the genetic background of common CVDs still have not been resolved: “Can genetic markers really improve CVD risk prediction? How many SNPs are responsible for the genetic component of CVD? How many genetic markers will we need to discover to reliably improve risk prediction? What are the implications of the allelic architecture of CVD for risk prediction? What necessary steps are needed before bringing this information to patients?” (123).

Although genetic screening/testing for CVDs would ideally offer proper options concerning prevention, the discriminatory power of genetic screening to identify those who should or should not be the target of specific lifestyle advice and/or specific medication is still controversial, especially for variants having low individual relative risk and low predictive values (18).

In conclusion, we found that only a small proportion of the genetic/genomic research has advanced from discovery phase to an evidence-based health application. But recent findings and especially GWA studies and prediction studies offer a more advanced level of primary/secondary prevention interventions for those subjects who are at greater genetic risk, hopefully in the near future. Presumably, developments in public health practice will also inevitably facilitate effective implementation of genomic science.

## Author Contributions

SF performed the literature search, interpreted the results, and wrote the manuscript. RÁ guided writing of the manuscript and was involved in finalizing the manuscript.

## Conflict of Interest Statement

The authors declare that the research was conducted in the absence of any commercial or financial relationships that could be construed as a potential conflict of interest.
